# Retraction Note: Transcriptional repression of SOCS3 mediated by IL-6/STAT3 signaling via DNMT1 promotes pancreatic cancer growth and metastasis

**DOI:** 10.1186/s13046-023-02595-3

**Published:** 2023-01-12

**Authors:** Li Huang, Bin Hu, Jianbo Ni, Jianghong Wu, Weiliang Jiang, Congying Chen, Lijuan Yang, Yue Zeng, Rong Wan, Guoyong Hu, Xingpeng Wang

**Affiliations:** Department of Gastroenterology, Shanghai General Hospital, Shanghai Jiaotong University School of Medicine, 100 Haining Road, Shanghai, 200080 Hongkou District China

**Retraction Note:** ***J Exp Clin Cancer Res*** **35, 27 (2016)**

**https://doi.org/10.1186/s13046-016-0301-7**

The Editor-in-Chief has retracted this article because of significant concerns regarding a number of irregularities that have been noted in figures 1C, 2A, 2C and 5A presented in this work, which question the integrity of the data. None of the authors have responded to any correspondence from the editor/publisher about this retraction.

